# Clinical applications of next‐generation sequencing‐based ctDNA analyses in breast cancer: defining treatment targets and dynamic changes during disease progression

**DOI:** 10.1002/1878-0261.13671

**Published:** 2024-06-12

**Authors:** Eva Valentina Klocker, Samantha Hasenleithner, Rupert Bartsch, Simon P. Gampenrieder, Daniel Egle, Christian F. Singer, Gabriel Rinnerthaler, Michael Hubalek, Katja Schmitz, Zsuzsanna Bago‐Horvath, Andreas Petzer, Sonja Heibl, Ellen Heitzer, Marija Balic, Michael Gnant

**Affiliations:** ^1^ Division of Oncology, Department of Internal Medicine Medical University of Graz Austria; ^2^ Institute of Human Genetics, Diagnostic and Research Center for Molecular BioMedicine Medical University of Graz Austria; ^3^ Division of Oncology, Department of Medicine I Medical University of Vienna Austria; ^4^ Third Medical Department with Hematology and Medical Oncology, Hemostaseology, Rheumatology and Infectious Diseases, Oncologic Center Paracelsus Medical University Salzburg Austria; ^5^ Department of Gynecology, Breast Cancer Center Tirol Medical University of Innsbruck Austria; ^6^ Department of Gynecology, Breast Cancer Center Vienna Medical University of Vienna Austria; ^7^ Department of Gynecology Breast Health Center Schwaz Austria; ^8^ Institute of Pathology University Medical Center Göttingen Germany; ^9^ Tyrolpath Obrist Brunhuber GmbH and Krankenhaus St. Vinzenz Zams Austria; ^10^ Department of Pathology Medical University Vienna Austria; ^11^ Department of Internal Medicine I for Hematology with Stem Cell Transplantation, Hemostaseology and Medical Oncology Barmherzige Schwestern, Elisabethinen, Ordensklinikum Linz GmbH Austria; ^12^ Department of Internal Medicine IV Klinikum Wels‐Grieskirchen GmbH Austria; ^13^ Institute of Human Genetics, Diagnostic and Research Center for Molecular BioMedicine, Christian Doppler Laboratory for Liquid Biopsies for early Detection of Cancer Medical University of Graz Austria; ^14^ Division of Hematology and Medical Oncology University of Pittsburgh School of Medicine PA USA; ^15^ Comprehensive Cancer Center Medical University of Vienna Austria

**Keywords:** breast cancer, circulating tumor DNA, liquid biopsy, precision medicine, targeted therapies, therapeutic targets

## Abstract

The advancements in the detection and characterization of circulating tumor DNA (ctDNA) have revolutionized precision medicine and are likely to transform standard clinical practice. The non‐invasive nature of this approach allows for molecular profiling of the entire tumor entity, while also enabling real‐time monitoring of the effectiveness of cancer therapies as well as the identification of resistance mechanisms to guide targeted therapy. Although the field of ctDNA studies offers a wide range of applications, including in early disease, in this review we mainly focus on the role of ctDNA in the dynamic molecular characterization of unresectable locally advanced and metastatic BC (mBC). Here, we provide clinical practice guidance for the rapidly evolving field of molecular profiling of mBC, outlining the current landscape of liquid biopsy applications and how to choose the right ctDNA assay. Additionally, we underline the importance of exploring the clinical relevance of novel molecular alterations that potentially represent therapeutic targets in mBC, along with mutations where targeted therapy is already approved. Finally, we present a potential roadmap for integrating ctDNA analysis into clinical practice.

## Graphical abstract

Circulating tumor DNA (ctDNA) offers a possibility for different applications in early and late stage breast cancer management. In early breast cancer tumor informed approaches are increasingly used for detecting molecular residual disease (MRD) and early recurrence. In advanced stage, ctDNA provides a possibility for monitoring disease progression and identifying resistance mechanisms. Figure created with BioRender.com.

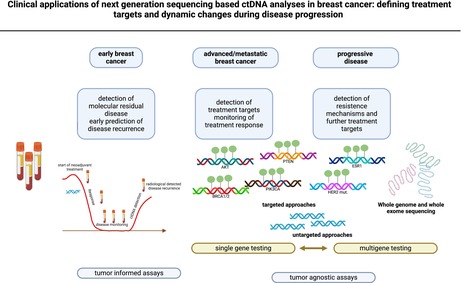

**Abbreviations**
AI: aromatase inhibitor
ASCO: American Society of Clinical Oncology
CDK4/6: cyclin‐dependent kinase 4/6
cfDNA: cell‐free DNA
CGP: comprehensive genomic profiling
ctDNA: circulating tumor DNA
ddPCR: digital droplet PCR
ER: estrogen receptor
ET: endocrine therapy
gBRCA1/2m: germline *BRCA1/2* mutations
HER2: human epidermal growth factor receptor 2
HR: hormone receptor
ICI: immune checkpoint inhibitor
mBC: metastatic breast cancer
mFAST‐SeqS: modified Fast Aneuploidy Screening Test‐Sequencing System
MRD: molecular residual disease
MSI: microsatellite instability
NCCN: National Comprehensive Cancer Network
NGS: next‐generation sequencing
ngSERD: next‐generation oral selective estrogen receptor antagonist and degrader
PARPi: poly ADP‐ribose polymerase inhibitor
pCR: pathological complete response
PFS: progression‐free survival
PR: progesterone receptor
RCB: Residual Cancer Burden
Safe‐SeqS: Safe‐Sequencing System
sBRCA1/2m: somatic *BRCA1/2* mutations
SERD: selective estrogen receptor degrader
SiMSen‐seq: simple, multiplexed, PCR‐based barcoding of DNA for sensitive mutation detection using Sequencing
SOC: standard of care
Tam‐Seq: tagged‐amplicon deep sequencing
TMB: tumor mutational burden
TNBC: triple‐negative breast cancer
TKI: tyrosine kinase inhibitor
VAF: variant allele fraction
WES: whole‐exome sequencing
WGS: whole‐genome sequencing

## 1 Introduction

The contemporary goal of precision medicine in patients with cancer is to identify biologically sensitive groups of tumors that will respond to targeted therapies. In this context, genomic sequencing of specific molecular alterations is increasingly performed in tissue samples, although tumor heterogeneity – both spatial and temporal – represents a major limitation of this approach [1]. An emerging alternative for capturing spatial sub‐clonal heterogeneity and clonal evolution during treatment, that allows for therapeutic clinical decision making, is the detection of circulating tumor DNA (ctDNA) via non‐invasive liquid biopsy [2, 3, 4].

Treatment choices in patients with breast cancer have historically been based on human epidermal growth factor receptor 2 (HER2) and hormone receptor (HR) status [5]. In the neoadjuvant setting, a major challenge is the prediction of response to systemic treatment before surgery in order to escalate treatment in non‐responders and de‐escalate treatment in responders, as appropriate [6, 7, 8, 9, 10]. To date, neither imaging methods including ultrasound [11] or MRI [12] nor image‐guided breast biopsies before surgery [13, 14, 15] have been able to adequately predict pathologic complete response (pCR). For this purpose, serial ctDNA analyses could provide a new approach to assess or predict tumor response early on during neoadjuvant treatment, thus ultimately guiding treatment decisions [10].

In metastatic BC (mBC), classical biomarkers such as HR and HER2 status are traditionally important for treatment decisions. Therefore, this review summarizes the status quo of the dynamic molecular characterization of mBC, especially in HR‐positive, HER2‐negative disease [16]. Moreover, we provide clinical practice guidance for the rapidly evolving field of molecular profiling of mBC and underline the importance of exploring the clinical relevance of novel molecular alterations that potentially represent therapeutic targets in mBC.

## 2 Tumor biology evolves over time

According to GLOBOCAN, in 2020, female breast cancer was the most commonly diagnosed cancer worldwide, with an estimated 2.3 million new cases and 685 000 deaths [17]. While breast cancer is mostly diagnosed at an early stage and associated with a 96% 5‐year survival probability in Europe [18], mBC still largely represents an incurable disease [19, 20]. Breast cancer can be subclassified into five intrinsic subtypes, that is, luminal A, luminal B, HER2‐enriched, basal, and normal‐like, which reflect fundamental differences at the molecular level and thus distinct clinical outcomes, mainly in patients with early breast cancer [21, 22, 23, 24].

Tumor (tissue) biopsy still represents the gold standard for diagnosis, classification, and treatment decisions. However, studies have found changes in subtypes at or after progression on anticancer therapies in up to 40% of tumors [25, 26, 27, 28, 29, 30, 31, 32, 33]. Importantly, beyond the HR and/or HER2 status detected in tissue, liquid biopsy provides a new concept to characterize and monitor the tumor genome and is increasingly being used as a tool to further guide clinical decision making [25, 29, 34, 35, 36].

After the diagnosis of metachronous metastatic (metastatic spread after 3 months after initial diagnosis) disease, patients often undergo initial confirmatory tissue biopsy including reassessment of the receptor subtype classification, which appears necessary due to a high rate of changes in HR and HER2 receptor status [25, 26, 27, 28, 29, 30, 31, 32, 33]. In addition, mBC could display major genomic differences compared to primary breast cancer as reported in sequencing studies of HR‐positive mBC [37]. In this context, the AURORA trial showed enriched alterations in several driver genes from metastatic lesions, including: *ESR1*, *PTEN*, *CDH1*, *PIK3CA* and *RB1* mutations, *MDM4* and *MYC* amplifications, *ARID1A* deletions, and clonality in genes like *RB1* and *ERBB2*. Additionally, high tumor mutational burden (TMB) was reported to correlate with shorter time to relapse in patients with HR‐positive/HER2‐negative mBC [29, 38]. Furthermore, in HR‐positive mBC, mechanisms of acquired resistance to prior endocrine therapy (ET) were linked to *ESR1* [39], MAPK pathway mutations [40, 41], and transcription factor mutations (e.g., *ARID1A* [40, 42, 43]). In contrast, in HER2‐positive and TNBC, no major genomic differences have been reported in primary vs. advanced settings [37, 40].

Although tissue may be analyzed using next‐generation sequencing (NGS), serial tissue biopsies are not routinely performed, given their invasiveness as well as anatomical constraints (e.g., bone metastasis). This further highlights the relevance of ctDNA analysis via NGS as an alternative. In this context, the phase 2a plasmaMATCH trial has paved the way for future personalized treatment approaches, since patients were selected for specific treatments based on the detection of mutations in ctDNA. Here, the sensitivity and specificity were comparable between tissue and ctDNA analysis for *AKT1*, *HER2*, and *PIK3CA* mutations [44]. Recently, we reported that elevated longitudinal trajectories of tumor fractions, as detected by the untargeted modified Fast Aneuploidy Screening Test‐Sequencing System (mFAST‐SeqS), were significantly associated with a higher progression risk in patients with HR‐positive/HER2‐negative mBC. The tumor agnostic, mFAST‐SeqS approach represents a simple and affordable method to estimate tumor fractions, that is, the proportion of cell‐free DNA (cfDNA) that is tumor‐derived ctDNA, by assessing chromosomal aneuploidy instead of mutations. After amplification and sequencing of uniquely mappable LINE1‐sequences across the genome, read counts are determined on a chromosome‐arm level and compared to a set of healthy controls. Deviations are represented as z‐scores and indicate gains and losses of chromosomal material. Finally, the squared sum of chromosome‐arm z‐scores is calculated, that is, the genome‐wide z‐score, which can be used as a surrogate for tumor fraction [45]. We have shown a very good correlation of mFAST‐SeqS based z‐scores with ‘ichorCNA’ tumor fractions [46]. Patients who developed progressive disease had higher baseline tumor fractions and showed constant increases over time. Furthermore, we have demonstrated the added value of evaluation of an untargeted assessment of tumor fractions along with cfDNA‐based mutational profiling [47].

## 3 Liquid biopsy

The broad concept of liquid biopsy encompasses the analysis of circulating nucleic acids, tumor cells, cell‐free RNA, extracellular vesicles, and tumor‐educated platelets that are released by primary or metastatic tumor lesions into the bloodstream or other body fluids. Thus, liquid biopsy represents a simple, non‐invasive method (e.g., based on blood sampling) providing a molecular footprint of the whole tumor entity [28, 35, 48, 49, 50, 51].

A number of processes involved in regular cell turnover, for example, necrosis or apoptosis, result in the release of cfDNA, whereas senescence prevents it [52, 53, 54, 55]. In the setting of cancer, a certain proportion of cfDNA is derived from tumor cells (i.e., primary and secondary tumor sites) and contains the full set of its genetic information. Thus, it is referred to as ctDNA [48, 49, 50, 51].

**Fig. 1:**
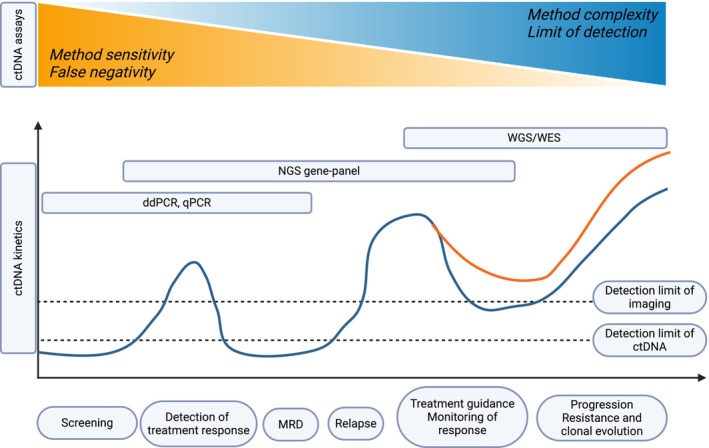
ctDNA dynamics along the clinical course of breast cancer. The blue line represents ctDNA variations over disease progression and the orange line represents the emergence of new mutations during treatment. ddPCR, digital droplet PCR; MRD, molecular residual disease; NGS, next‐generation sequencing; qPCR, quantitative PCR; WES, whole‐exome sequencing; WGS, whole‐genome sequencing. Adapted from [142]. Figure created with BioRender.com.

The amount of ctDNA ranges from 0.01% to 0.1% in early‐stage to 5–10% or even higher in advanced‐stage cancers where cancer cells are more abundant and undergoing more rapid cell division [51, 56]. While both cfDNA and ctDNA can include fragments of similar sizes, ctDNA fragments often exhibit a more pronounced peak at shorter lengths or a more diverse size distribution [57]. ctDNA varies with respect to fragment length, numbers, and variant allele frequency (VAF) of alterations and has a very short half‐life of < 120 min [58], despite the lack of comprehensive pharmacokinetic studies to accurately establish this. ctDNA assessment allows for a comprehensive genomic snapshot of the tumor mutational content, even in cases of tumor heterogeneity and cell plasticity during cancer progression [58, 59, 60]. The ctDNA dynamics along the clinical course of breast cancer including appropriate ctDNA assays and their sensitivity are depicted in Fig. 1.

## 4 Preanalytical and analytical considerations

Plasma seems to be the ideal analyte for ctDNA collection and diagnostic purposes in solid tumors, including breast cancer [48, 49, 50]. There is a consensus to use specialized collection tubes, for example, PAXgene Blood circulating cfDNA tubes or those from commercial providers that contain an additive to stabilize blood cells, thus preventing cell lysis, particularly if plasma extraction cannot be done with 4–6 h after blood draw. After plasma separation, DNA extraction is performed using commercial kits specifically designed for cfDNA, and finally, the amount of cfDNA is quantified [61]. Importantly, the selection of the approach to library preparation dictates the final type of information obtained from the analysis.

Although there are several targeted sequencing approaches available, the most common ones are amplicon‐based (i.e., tagged‐amplicon deep sequencing (TAm‐Seq), Safe‐Sequencing System (Safe‐SeqS), and simple, multiplexed, PCR‐based barcoding of DNA for sensitive mutation detection using Sequencing (SiMSen‐seq)), and hybrid capture‐based (i.e., pan‐cancer ctDNA expanded liquid biopsy panel) methods. Briefly, the PCR amplicon‐based method uses specific primers to amplify and enrich only target genomic regions of interest. After amplification, sequencing adapters and barcode indexes are added to create the library for sequencing. In the hybrid capture method, genomic DNA is first fragmented followed by ligation of adapters and barcode indices to generate a genomic library which is further subjected to hybridization using long, biotinylated specific oligonucleotide baits (probes). The target sequences are captured using streptavidin magnetic beads followed by elution of the library which is further subjected to PCR amplification and deep sequencing [61, 62]. Overall, amplicon‐based sequencing requires lower amounts of DNA input, is generally more cost‐effective, and has higher on‐target rates. As a result, it is more often used in small‐scale experiments. In contrast, hybridization capture provides more uniform coverage of on‐target reads. The longer bait sequences allow for a greater level of specificity in region selection. Furthermore, it produces fewer PCR duplicates, making it particularly useful for samples where PCR artifacts are likely to occur, such as ctDNA samples. Therefore, it is the preferred methodology for large panels and whole‐exome sequencing [62, 63].

## 5 Choosing the right NGS‐based ctDNA assay

The choice of the right assay, which may be based on PCR, ddPCR, or NGS, first depends on the clinical question at hand, what type of information needs to be harvested from the sample, and the disease stage of the patient. In terms of NGS‐based approaches, the decision generally relies on the distinction between a targeted approach that focuses on particular predefined regions of the genome and an untargeted approach, such as whole‐exome sequencing (WES), which targets all human protein coding genes, or whole‐genome sequencing (WGS). Moreover, these assays can either be personalized based on existing sequencing data or tumor‐agnostic, which means that the assessment is performed without any prior knowledge of the tumor genome (or in the absence of any baseline data) [61].

In the setting of early disease and MRD (molecular residual disease) monitoring, where the likelihood of detecting ctDNA fragments must be maximized, tumor‐informed NGS assays designed to track patient‐specific mutations longitudinally are typically used, as they can achieve the required sensitivities to detect minute traces of ctDNA [64, 65, 66]. This approach entails WES or WGS of a baseline tissue or plasma sample and then subsequently designing patient‐specific panels to screen for dozens or up to thousands of mutations. These approaches enable the detection of ctDNA at levels below one part‐per‐million (< 0.0001%) (ppm) [66]. However, a variety of tumor‐agnostic approaches employing multi‐omics methods, such as ctDNA methylation or fragment‐omics, have also demonstrated promising performances, and many more are in development [67].

For patients with advanced cancer, the most predominant current clinical use case for ctDNA analysis is molecular profiling for the selection of targeted treatments. In this regard, the strategy is to maximize the identification of actionable alterations that can be matched to targeted therapies. Typically, pan‐cancer comprehensive genomic profiling (CGP) panels are employed to enable the detection of many clinically relevant biomarkers [61].

There are several commercially available kits enriching for tens or hundreds of genes. For example, the AVENIO ctDNA Expanded kit, that is based on the Cancer Personalized Profiling by deep Sequencing (CAPP‐Seq) [68], contains 77 genes, of which 17 biomarkers are recommended by the National Comprehensive Cancer Network (NCCN) guidelines and 60 biomarkers are currently under investigation in clinical trials. Other panels, such as the TruSight Oncology 500 ctDNA which enables in‐house CGP of ctDNA and targets 523 genes to assess DNA variants across major variant classes (SNV, MNV, indels, CNV, and gene rearrangements), also include the assessment of TMB or microsatellite instability (MSI). If there are no in‐house partner laboratories that carry out such analyses, samples can be sent to a commercial end‐to‐end provider to provide the molecular tumor board with all relevant information [61].

**Fig. 2:**
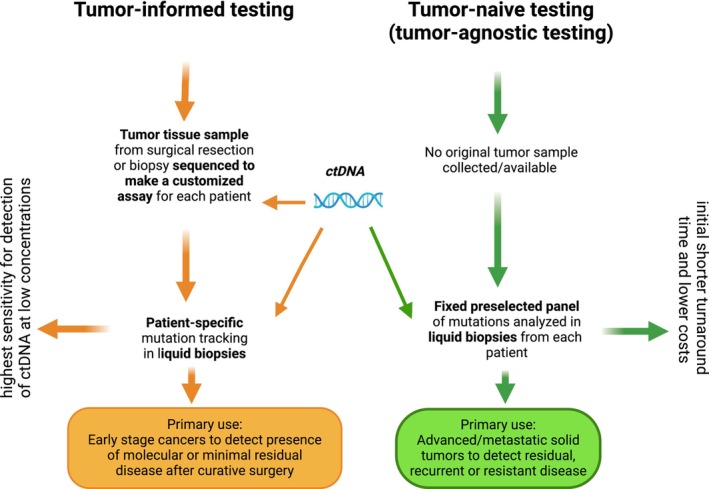
Tumor‐informed vs. tumor‐naive approaches. This figure gives an overview of strategies for liquid biopsy based in clinical practice. In orange the applications of tumor‐informed assays are highlighted, in green the use of tumor‐naïve assays is shown. Adapted from [143, 144]. Figure created with BioRender.com.

It is also worth mentioning that other ctDNA‐based approaches may have specific applications that do not require NGS. For example, the Therascreen® PIK3CA RGQ PCR kit, which received FDA approval for advanced‐stage HR‐positive/HER2 ‐negative breast cancer based on the findings of the phase III SOLAR‐1 trial, is a real‐time PCR test that screens for 11 therapy‐relevant *PIK3CA* mutations from plasma [69]. Tables 1, 2, 3 summarize available ctDNA assays to direct therapy in advanced disease, for MRD detection and early detection, respectively, while Fig. 2 depicts ctDNA analytical approaches.

**Table 1:** Examples of commercially available ctDNA assays to direct therapy in advanced disease. WBC, white blood cells.

Test name	Company	FDA approval as companion diagnostic	Number of genes in panel	Complex biomarkers included	Trials performed with the assay
Foundation Liquid CDx	Foundation Medicine	Yes	324	Yes	IMAGE trial mTNBC, ≥ 1 treatment lines, genomic driven therapies, NGS in tissue vs. plasma [76]
Guardant360 CDx	Guardant Health	Yes	74	Yes	mBC, ctDNA ESR1 status for treatment with Elacestrant (Emerald trial [77]) *ESR1*, *HER2, AKT1, PTEN* mutations for targeted therapies (plasmaMATCH [78])
PGDx elio Plasma Resolve	Labcorp	No, but has received Breakthrough Device Designation	33	No	Metastatic colorectal cancer, ctDNA in combination with WBC for treatment response monitoring [79]
PGDx elio Plasma Complete	Labcorp		521	Yes	–
Tempus xF+	Tempus	No	523	Yes	mBC NGS is tissue vs. ctDNA [80]
Circulogene TumorClear	Circulogene	No	88	Yes	–

**Table 2:** Examples of commercially available ctDNA assays for MRD detection. Adapted from [74].

Test name	Company	FDA approval	Tumor types	Details	Trials
RaDAR	NeoGenomics	No, but received Breakthrough Device Designation	Breast, colon, lung, head & neck	Tumor whole‐exome sequencing (WES) and monitoring of up to 48 tumor/patient‐specific mutations	High‐stage II‐III HR‐positive mBC, ctDNA to detect MRD and development of metastasis [81]
Guardant Reveal	Guardant Health	No	Early‐stage (II&III) CRC	Tumor‐uninformed approach, integrates somatic alterations with an epigenomic cancer signature to identify the presence of methylation signatures associated with cancer *vs*. normal DNA	Colorectal Cancer To detect MRD and correlation with recurrence [82]
brPROPHET™	Burning Rock Biotech	No	Early‐stage CRC	Tumor WES followed by customized design of a patient‐specific panel consisting of 50 single‐nucleotide variants	Colorectal Cancer Stages I–IV, detection of MRD [83]
PCM	ArcherDx/Invitae	No	NSCLC	Tumor WES followed by customized design of a patient‐specific panel consisting of 50 single‐nucleotide variants	NSCLC Stages I–III Detection of MRD and correlation with relapse [84]
Signatera™ Residual disease test (MRD)	Natera™	No, but received Breakthrough Device Designation	Colorectal, NSCLC, breast, and bladder cancer	Tumor WES of tissue sample and matched normal and selection of 16 patient‐specific, clonal somatic variants for design of custom 16‐plex PCR followed by NGS to monitor ctDNA longitudinally. A positive Signatera™ result predicts relapse with overall positive predictive value more than 98%	High‐risk early stage HER2‐ negative BC Detection of ctDNA clearance under neoadjuvant treatment, correlation of response, lack of ctDNA clearance was associated with reduced distant free survival [85]
Haystack MRD™	Quest Diagnostics™	No	Solid tumors	Tumor WES to identify individual‐specific mutations and selection of up to 50 to personalize the subsequent MRD assay.	Colorectal Cancer, Stage II 1 : 2 randomization Intervention cohort: treatment was only given when positive ctDNA at week 4 or 7 was detected, recurrence‐free survival was not inferior to standard cohort [86]

**Table 3:** Examples of commercially available ctDNA assays for early cancer detection.

Test name	Company	FDA approval	Tumor types	Details
CancerSEEK	Exact Sciences	No	Multiple	A multi‐biomarker class (mutations, methylation, proteins, and aneuploidy) and machine‐learning approach. CancerSEEK was used in DETECT‐A study [87]. Test is under further development. The features above describe current development goals
Epi proColon	Epigenomics	Yes	Colorectal cancer	Real‐time PCR detection of Septin9 methylation, which is altered in colorectal cancer tumor cells more often than in normal cells [88]
Epi proLung	Epigenomics	No	Lung cancer	Real‐time PCR assay for the qualitative detection of SHOX2 and PTGER4 gene methylation in DNA [89]
Shield	Guardant Health	No, but submitted application for Pre‐Market Approval	Colorectal cancer	Uses a multimodal approach, integrating genomics, epigenomics, and proteomics, to achieve high sensitivity and specificity in detecting early signs of colorectal cancer in average‐risk adults aged 45 and older (ECLIPSE: NCT04136002)
Galleri®	GRAIL	No	Multi‐cancer (50+ types)	Plasma cfDNA bisulfite sequencing targeting a panel of > 100 000 informative methylation regions. Cancer detection and tissue of origin (TOO) localization through a proprietary classifier. Recently tested in the large‐scale SYMPLIFY study in a symptomatic patient population [90]

## 6 Early breast cancer – risk stratification and early assessment of disease progression

In patients with early breast cancer, ctDNA levels were shown to be elevated before and after surgery compared to those with benign breast lesions, and ctDNA levels correlated with nodal involvement and tumor size [82]. Additionally, detection of ctDNA during follow‐up in patients with early breast cancer was shown to be associated with a high risk of future relapse [83]. In terms of response rates to neoadjuvant systemic treatment and pCR, studies have shown lower rates of pCR in patients where ctDNA was detected before the administration of neoadjuvant anti‐HER2 therapies [84]. In a subanalysis of the NEOALLTO trial, ctDNA detection before neoadjuvant treatment was significantly associated with older age and ER negativity. The lowest pCR rate was detected in patients with detectable ctDNA before and during neoadjuvant treatment, whereas during neoadjuvant treatment, a rapid decrease in ctDNA levels (ctDNA clearance) was linked to response like pCR [85, 86]. Moreover, the detection and persistence of ctDNA during neoadjuvant systemic treatment was shown to predict poor response [10] and metastatic recurrence [87]. Presence of ctDNA towards the end of treatment is expected to reflect residual disease [74]. Magbanua et al. [76] evaluated ctDNA clearance under neoadjuvant treatment in 295 patients with early disease with the Signatera™ residual disease assay, a personalized tumor‐informed ctDNA test. Early ctDNA clearance was significantly associated to Residual Cancer Burden (RCB) 0 and pCR, respectively, in triple negative breast cancer (TNBC) but not for HR‐positive and HER2‐positive tumors. ctDNA persistence at the time of surgery was significantly associated with increased risk of relapse and worse overall survival. However, the data demonstrate that particularly in HR‐positive patients more sensitive tests are needed to decrease the false‐negative cases. Further clinical data are needed to establish the value of ctDNA in these groups. Nevertheless, this trial is so far the largest neoadjuvant trial demonstrating the potential of ctDNA testing at an early neoadjuvant stage. Another trial by Parson et al. [70] enrolled solely TNBC and demonstrated that tumor‐informed clearance of ctDNA was associated with pCR and RCBI or 0 and that five of six patients with relapse were ctDNA positive before surgery.

In patients with non‐metastatic (stage I–III) breast cancer, serial monitoring of ctDNA detected progression with a sensitivity of 86–93% and a specificity of 100% with an average lead time of 11 months (range, 0.5–37) even before recurrent disease was detected by imaging or clinical signs became apparent [75, 88, 89].

Despite these promising data, the primary concern is still the restricted sensitivity of existing assays in accurately identifying all patients at risk of relapse. Consequently, studies investigating the potential for treatment de‐escalation based on ctDNA are not yet poised for initiation. However, findings from all studies conducted in the neoadjuvant setting thus far, including our own research, consistently indicate that the persistence of ctDNA under neoadjuvant treatment carries an unfavorable prognosis. Early assessment in these cases may serve as a basis for treatment intensification, thus enhancing the prognosis for these patients. Many follow‐up studies evaluating ctDNA detection after treatment are underway. Turner et al. [90] examined a surveillance strategy and advocated for treatment escalation among patients with TNBC based on ctDNA positivity. In the intervention cohort, patients received pembrolizumab when ctDNA was detected. However, with only nine of 32 patients remaining in the intervention cohort after staging, and only five receiving pembrolizumab, the trial was underpowered and enriched for high‐risk patients. Only one patient experienced a drop in ctDNA levels under treatment. Many similar trials evaluating ctDNA detection after treatment are underway and we anticipate a substantial rise in evidence, with several methods outlined in Tables 1, 2, 3 showing encouraging outcomes for future clinical interventions [73, 91, 92].

## 7 Advanced breast cancer – detection of an increasing number of genome‐based therapeutic targets

While ctDNA testing may be used to identify disease relapse prior to imaging in early‐stage breast cancer as a future perspective, it is already becoming a crucial part of routine clinical practice in mBC. This is due to the high number of actionable mutations that can be detected, as well as the utility of ctDNA as a surrogate for tumor burden. Besides ER, progesterone receptor (PR), and HER2 status, there is an increasing number of other biomarkers known to predict drug benefits and affect patient management and treatment approaches [93, 94]. These include germline *BRCA1/2* mutations (g*BRCA1/2*m) in HER2‐negative mBC, PD‐L1 in TNBC or PIK3CA in ER/PR‐positive, HER2‐negative mBC [95] and, more recently, *ESR1* mutations. In this context, the development of endocrine resistance in mBC represents a challenging clinical scenario in which serial testing of ctDNA represents a vital tool for early detection of resistance mechanisms and prediction of progression‐free survival (PFS) [96, 97, 98].

In patients with locally advanced or metastatic ER‐positive/HER2‐negative breast cancer, ET with either aromatase inhibitors (AI) or fulvestrant plus a cyclin‐dependent kinase 4/6 (CDK4/6) inhibitor is recommended as first‐line standard of care (SOC). Progression after at least one line of ET or CDK4/6 inhibitor therapy is often associated with endocrine resistance, which includes development of acquired mutations in a variety of genes, including *ERBB2*, *NF1*, and estrogen receptor 1 *ESR1* [95, 99].

### 7.1 *ESR1* mutations

**Fig. 3:**
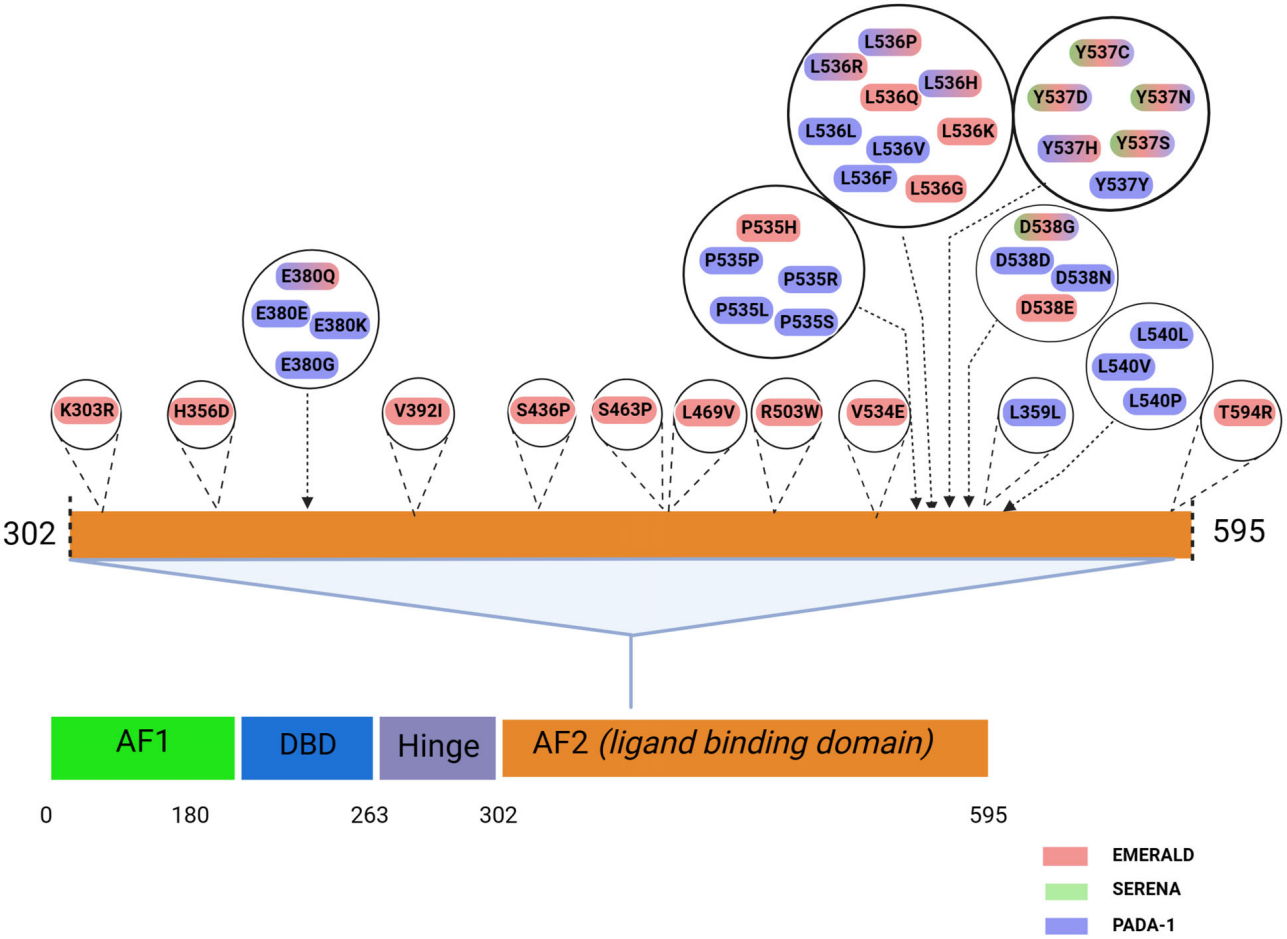
List of currently clinically relevant ESR1 mutations in patients with endocrine resistant ER‐positive breast cancer (as analyzed in the current studies leading to approval or upcoming approval of SERDs in. development). ER, estrogen receptor; SERD, selective estrogen receptor degrader. Figure created with BioRender.com.

Mutations in the *ESR1* gene that encodes for ERα are known drivers of resistance to first‐line AI, and approximately 40% of these patients acquire *ESR1* mutations over time during the disease progression and evolution [100, 101, 102]. The most common *ESR1* hotspot mutations are Y537S/N/C, D538G, and L536Y (see Fig. 3) [103]. A combined analysis of the phase III SoFEA and EFECT Trials, which enrolled HR‐positive mBC patients, demonstrated that patients with *ESR1* mutation at baseline who had previously progressed on nonsteroidal AI therapy experienced inferior PFS and OS when treated with exemestane vs. fulvestrant 250 mg [72]. The PALOMA‐3 trial further investigated specific hotspot mutations, some of them assumed to be sub‐clonal in resistant cancer cells. Specifically, in 114 of 445 patients an ESR1 hotspot mutation was detected; and in 100 patients, a PIK3CA hotspot mutation was found at treatment initiation. On day 15 of treatment, ctDNA levels of ESR1 mutations and also the levels of PIK3CA mutations significantly declined, especially in the cohort receiving palbociclib. To measure the molecular response of ctDNA the ratio of mutant copies·mL^−1^ of plasma was compared between day 1 and day 15. In contrast to *PI3KCA*, the early dynamics of *ESR1* mutations failed to predict PFS in this trial. Although there was a decrease in *ESR1* mutant clones upon treatment with fulvestrant ± palbociclib, this did not correlate with long‐term improvement in PFS [104]. Nevertheless, one could speculate that decreasing levels of ctDNA correlated with some response to treatment, a concept which is now also evaluated in prospective clinical trials.

The PADA‐1 trial adopted a pioneering strategy by exchanging the endocrine partner of CDK4/6 inhibitor therapy upon the detection of an *ESR1* mutation in patients with ER‐positive/HER2‐negative mBC, which led to almost doubling the PFS by switching from AI + palbociclib to fulvestrant + palbociclib [105]. A multiplex digital droplet PCR (ddPCR) based detection of ESR1 mutations was conducted analyzing only a subset of ESR1 hotspot mutations (Fig. 3). *ESR1* mutations result in estrogen‐independent ER activation and thus, resistance to AI, but not to ER inhibitors, arises [100, 106]. However, as recent trials with oral selective estrogen receptor degraders (SERDs) have shown, fulvestrant has less activity in patients with *ESR1* mutations. This was particularly shown in the SERENA 2 trial, where ctDNA‐based ESR1 clearance was earlier and more effective with camizestrant as with fulvestrant. The concept of PADA1 trial was novel, although the testing strategy in future trials may be improved, and treatments selection toward more effective strategies may be more appropriate. Currently ongoing clinical trials address this question. The EMERALD trial evaluated the efficacy and safety of the oral ERD elacestrant vs. standard‐of‐care ET (either AI or fulvestrant) in previously treated patient with ER‐positive/HER2‐negative mBC Elacestrant treatment resulted in a 30% reduction in the risk of progression or death compared to SOC in the overall cohort, as well as a 45% risk reduction in patients with *ESR1* mutation [71]. However, some patients harboring *ESR1* mutations do not benefit from elacestrant, which is indicated by an initial drop in PFS that may indicate ‘total endocrine resistance’ [71]. Therefore, it is important to identify further biomarkers for the selection of patients who will benefit from other treatment regimens, for example, chemotherapy or antibody drug conjugates. Whereas the EMERALD trial has been the first which led to approval of an oral SERD in palliative treatment, only a small proportion of patients will be selected for single agent treatment after progression on combinations with CDK4/6 inhibitors. Nevertheless, these data gave rise to an update of the American Society of Clinical Oncology (ASCO) guidelines that now include a testing recommendation for *ESR1* mutations, as well as a recommendation of the oral SERD elacestrant for patients with mBC and detectable *ESR1* mutations who experience disease progression on ET with or without prior CDK4/6 inhibitor treatment. Moreover, the updated ASCO guidelines state that retesting at subsequent disease progression is warranted to determine whether an *ESR1* mutation has emerged [107]. The FDA has already approved elacestrant and the Guardant360 CDx assay as a companion diagnostic device in the beginning of 2023. The EMA has approved elacestrant in September 2023 for the treatment of postmenopausal women and men with ER‐positive/HER2‐negative, locally advanced, or mBC with an activating *ESR1* mutation who have disease progression following at least 1 line of ET including a CDK4/6 inhibitor. This implicates an urgent need for reliable *ESR1* testing.

In the SERENA‐2 trial, camizestrant, a next‐generation oral selective estrogen receptor antagonist and degrader (ngSERD), is being studied with promising results in post‐menopausal women with advanced ER‐positive/HER2‐negative BC. At the San Antonio Breast Cancer Symposium, Oliveira et al. [108] showed that treatment with camizestrant (both 75 and 150 mg) resulted in eradicated or near undetectable *ESR1m* ctDNA levels by day 1 of cycle 2. These lower levels were maintained for at least 6 months. These data suggest that an early intervention upon detection of an *ESR1* mutation may provide the maximum patient benefit, as shown by a PFS of 12.9 months (range, 4.5–14.7) on camizestrant treatment vs. 2.2 months (range, 1.9–9.3) in fulvestrant‐treated patients with only a single *ESR1* mutation [109]. This is being investigated further in the randomized, double‐blind, phase III SERENA‐6 trial. In that trial, the efficacy and safety of switching from an AI to camizestrant is assessed in patients with HR‐positive/HER2‐negative mBC on first‐line therapy upon detection of an *ESR1* mutation in ctDNA, while maintaining treatment with the same CDK4/6 inhibitor [110]. In addition, these data underline the potential of ctDNA as a monitoring tool for treatment response.

Another approach to overcome the limited treatment options for patients with mBC with *ESR1* mutations is lasofoxifene, a third‐generation selective estrogen receptor modulator. In the ELAINE‐1 study, lasofoxifene demonstrated antitumor activity compared to fulvestrant [111], and in ELAINE‐2, lasofoxifene showed antitumor activity in combination with abemaciclib [112]. Based on these data, patients with *ESR1*‐mutant, ER‐positive, HER2‐negative mBC will be randomized to either abemaciclib plus fulvestrant or abemaciclib plus lasofoxifene in the large, randomized phase III ELAINE‐3 trial (NCT05696626) that is using the FDAapproved Guardant360® CDx liquid biopsy NGS–based test as a companion diagnostic for the sequencing of 74 genes. Although the clinical need for liquid biopsy‐based testing is becoming increasingly apparent, none of the assays described or listed here are currently reimbursed in a routine clinical setting in Austria, where the authors are actively practicing. This applies to all mutations and targets discussed, including *PIK3CA* or *ESR1* hotspot mutations, among others. The situation is particularly crucial for ESR1 testing, since the authors mandate ctDNA‐based detection of *ESR1* mutation in order to reimburse elacestrant. Thus, there is no clear recommendation for a specific assay to be used, but rather an attempt of the authors to address this critical issue.

In addition to detecting *ESR1* point mutations, liquid biopsies were shown to efficiently identify *ESR1* fusions, particularly in metastatic ET‐resistant ER‐positive breast cancer [113]. Since not all *ESR1* fusions are clinically actionable, Gou et al. [114] developed a 24‐gene expression signature specific for transcriptionally active *ESR1* fusion proteins. Depending on the ESR1 fusion type, there are different strategies, for example, targeting of CDK4/6 if an active ligand‐binding/Activation Function 2 domain swap is detected, or downstream CDK4/6 inhibition if the ligand‐binding domain is absent, or administration of HER2‐directed antibody‐drug‐conjugates if a patient presents with an ESR1::CCDC170 fusion [115]. However, these are observations from case studies, and further strategies aimed at precision treatment of these resistance alterations is warranted.

### 7.2 *PIK3CA* mutations

In 28–46% of patients with HR‐positive/HER2‐negative mBC, mutations in the *PI3KCA* gene that encodes the p110α subunit of phosphatidylinositol‐3‐kinase (PI3K) are reported and these are associated with chemoresistance and poor prognosis [116, 117, 118]. The SOLAR‐1 trial that included HR‐positive/HER2‐negative mBC patients showed that the addition of alpelisib, an orally bioavailable α‐selective PI3K inhibitor, resulted in a significant and clinically meaningful PFS benefit in the presence of the *PIK3CA* mutation regardless of its location (E542X, E545X, or H1047X). Although the overall survival results did not cross the prespecified efficacy boundary, a 7.9‐month numeric improvement in median overall survival was reported in patients receiving fulvestrant plus alpelisib [118]. In the BYLieve trial, alpelisib plus fulvestrant also showed a significant impact on PFS in patients who had experienced progression on a CDK4/6 inhibitor plus an AI [119]. We have shown that the SiMSen‐Seq‐based detection of PIK3CA mutations in plasma exhibits advantageous concordance with the tissue analyses, and when combined with an untargeted, mutation‐independent approach for detecting ctDNA fractions, it enhances the performance of the SiMSen‐Seq test. This combinatory approach helps select suitable patients for alpelisib treatment [120]. Our current approaches are focusing on maintaining the sensitivity for *PIK3CA* mutational testing while also allowing for testing of additional targets including *ESR1*.

### 7.3 Protein kinase B ( *AKT* ) mutations

The protein kinase B (AKT) comprises three highly homologous isoforms: namely Akt1 (PKBα), Akt2 (PKBβ), and Akt3 (PKBγ) [121]. In addition to *PIK3CA* and *ESR1*, *AKT* is implicated in endocrine resistance in HR‐positive/HER2‐negative mBC. The phase III CAPItello‐291 trial investigated the efficacy and safety of the pan‐AKT inhibitor capivasertib + fulvestrant in patients with AI‐resistant HR‐positive/HER2‐negative mBC [122]. The dual primary endpoint consisting of investigator‐assessed PFS in the overall population (7.2 vs. 3.6 months) as well as in patients with AKT pathway‐altered tumors (7.3 vs. 3.1 months) was met. Besides *PIK3CA* mutations, *AKT1* and *PTEN* mutations were associated with greater benefit from capivasertib then in the overall population. Although NGS was performed in tumor tissue [123], other trials have already identified *AKT1* and *PTEN* mutations via ctDNA and allocated patients to an appropriate therapy [44]. Nevertheless, if included in the panel, this information may add information to clinical interpretation.

### 7.4 ERBB2 (HER2) mutations

Somatic activating mutations in the *ERBB2* (*HER2*) gene are present in approximately 3–5% of patients with metastatic HR‐positive breast cancer and are associated with endocrine resistance due to crosstalk between HER2 and ER signaling pathways. In the SUMMIT trial, neratinib, an oral, irreversible, pan‐HER tyrosine kinase inhibitor (TKI), was administered in combination with fulvestrant and trastuzumab to patients with HR‐positive *HER2*‐mutant mBC after progression on CDK4/6 inhibitor therapy. The triplet combination showed encouraging clinical efficacy with an overall response rate of 39% (95% CI, 26–52) and a median PFS of 8.3 months (95% CI, 6.0–15.1) [124]. The value of liquid biopsy‐based detection of *HER2* mutation was clearly suggested by plasmaMATCH trial [44].

### 7.5 *BRCA* mutations

Approximately, two thirds of the *BRCA1/2* mutations found in breast cancer are germline mutations, whereas the remaining third are somatic [125]. While the efficacy of poly ADP‐ribose polymerase inhibitor (PARPi) therapy is currently under investigation for mBC with somatic *BRCA1/2* mutations (sBRCA1/2m) by the Translational Breast Cancer Research Consortium (NCT03344965), there are data on germline mutations in *BRCA1* or *BRCA2* already, which are carried by approximately 5% of unselected patients with breast cancer [126] and are detected in up to 20% of patients with TNBC [127]. The findings of the phase III OlympiAD trial (olaparib vs. chemotherapy median PFS of 7.0 vs. 4.2 months; hazard ratio = 0.58, 95% CI: 0.43–0.80) showed that patients with *HER2*‐negative locally advanced breast cancer or mBC and g*BRCA1/2m* derive significant benefit from PARPi therapy, which also applied to the phase III EMBRACA study (talazoparib vs. chemotherapy, median PFS of 8.6 vs. 5.6 months; hazard ratio = 0.54, 95% CI: 0.41–0.71) [128, 129]. An investigator‐initiated phase II study revealed that olaparib is also effective in patients with s*BRCA1/2m*, with almost similar ORR (50%, 90% CI: 28–72) as reported in the OlympiAD and EMBRACA trials (59.9%, 95% CI: 52.0–67.4 and 62.2%, 95% CI: 55.8–69.0, respectively) and a median PFS of 6.3 months (90% CI, 4.4–not available) [128, 129, 130]. It is of importance that the majority of used NGS panels incorporate *BRCA* mutations, and in most cases, the allele frequency of mutations enables conclusions regarding the origin of mutations (germline vs. somatic). Also, other genes that play an important role in the management of patients with mBC, including *TP53*, *GATA3*, and *ARID1A* can be assessed. However, since these are not yet clinically established, we did not cover these alterations in detail [131].

## 8 TMB and MSI as predictive complex biomarkers for immunotherapy

High TMB defined as ≥ 10 mutations per megabase, leads to clinically meaningful benefit from treatment with pembrolizumab in several cancer types [132]. However, no patients with mBC had been included in this study. However, in an explorative analysis from the Impassion130 trial, higher TMB was associated with better OS in the PD‐L1 positive subgroup [133]. In both trials, TMB was tested in tumor tissue. It was shown that TMB in plasma, referred to as blood TMB, is highly correlative to tissue‐based TMB. However, data in breast cancer regarding blood TMB as a predictive marker for immune checkpoint inhibitor (ICI) are lacking. MSI is another biomarker for the clinical benefit of ICI [134]. Also, prospective data demonstrating liquid biopsy‐based detection of MSI for the prediction of utility of ICI may be promising. The assessment of these theragnostic markers – TMB and MSI – demonstrated feasibility in ctDNA assays, although studies in patients with breast cancer are still limited [135]. A list of mutations and matched therapies according to FDA and EMA approval is displayed in Table 4.

**Table 4:** Mutation diagnosis – precision medicine for mBC targeted therapies.

Genomic finding	Matched therapy
*ESR1* ^mut^	Elacestrant* ^,^ ^†^
*PIK3CA* ^mut^	Alpelisib (+ fulvestrant)* ^,^ ^†^
*HER2* ^mut^	Neratinib* ^,^ ^†^, Lapatinib* ^,^ ^†^
*BRCA1/*2	Olaparib* ^,^ ^†^, Talazoparib* ^,^ ^†^
*NTRK* fusion	Larotrectinib* ^,^ ^†^, Entrectinib* ^,^ ^†^
Microsatellite instability‐high or mismatch repair deficient	Pembrolizumab* ^,^ ^†^
Tumor mutational burden‐high ≥10 mut/Mb	Pembrolizumab* ^,^ ^†^
PDL‐L1 expression ≥1%	Atezolizumab* ^,^ ^†^
PD‐L1 combined positive score ≥ 10	Pembrolizumab* ^,^ ^†^
*RET* fusion	Selpercatinib*

*FDA approved.

† EMA approved.

## 9 Outlook and considerations

**Fig. 4:**
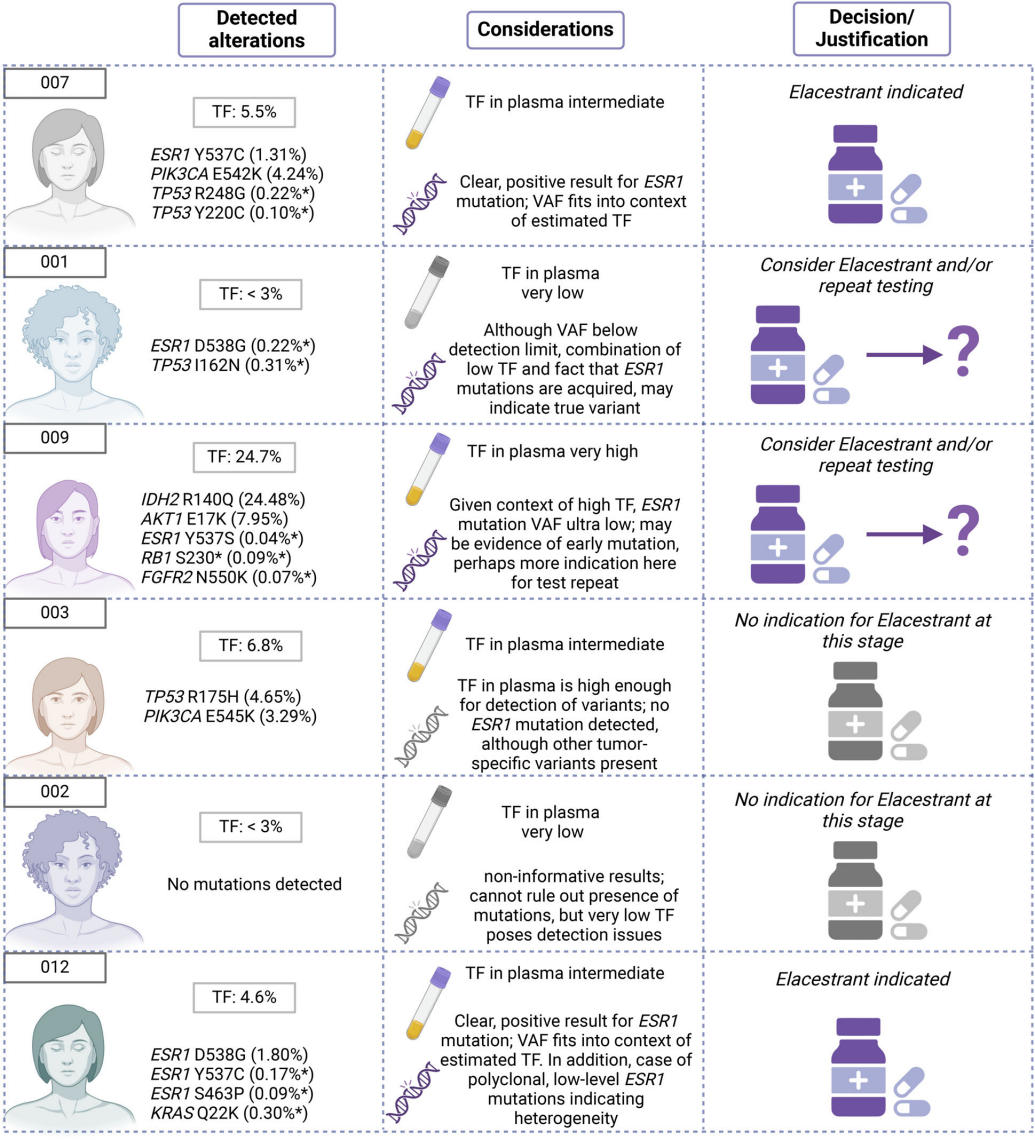
Six examples for patients with mBC with *ESR1* molecular testing results. Six patient examples are illustrated to demonstrate the spectrum of results that may be observed from routine molecular profiling of plasma DNA in the clinical setting. The same 77‐gene CGP panel was employed for all cases. This assay has been validated to reliably detect variants down to a VAF of 0.5%, indicating that any variant with a VAF below this percentage is below the technical LOD. In the left‐most panel, the alterations detected are listed alongside their VAFs, which are provided as a percentage in parentheses. An asterisk next to the VAF designates that this variant is below the assay LOD. In addition, the TF in plasma was estimated with a standard algorithm (ichorCNA), which has an LOD of 3%, and is provided in the box above the list of detected variants. In the middle column, a brief orientation and interpretation of the results is provided, which should help understand the technical and biological considerations that play a role in the clinical decision. The third column provides a brief justification of the treatment implications. As an official guideline or decision tree for the implementation of *ESR1* mutation testing results is currently lacking, the justification here serves as more of a high‐level interpretation of the results for clinicians. Importantly, this highlights the utility of the molecular tumor board in performing an interdisciplinary, comprehensive evaluation of the results within the individual patient context to derive the most evidence‐based decision. CGP, comprehensive genomic profiling; LOD, limit of detection; mBC, metastatic breast cancer; TF, tumor fraction; VAF, variant allele frequency. Figure created with BioRender.com.

Although the indications and guidelines for molecular profiling of patients with mBC outlined above appear straightforward, integrating molecular testing results from ctDNA into routine clinical practice requires a careful understanding of the associated technical caveats and clinical implications, which can differ among clinics. In terms of the test itself, it is recommended to utilize a well validated assay with known performance metrics, ensuring that molecular findings can be accurately interpreted [136]. In addition, the molecular test must align with the therapy indication, costs, and reimbursement thereof, which plays a major role in the selection of the assay. In this context, a genomic testing cost calculator has demonstrated that the use of NGS for up to 50 genes compared to sequential single‐gene testing (PCR for *PIK3CA*, IHC for HER2 amplification and MSI‐H, as well as FISH for *NTRK* fusions) led to a cost reduction of approximately 42% per correctly identified patient with mBC [137]. Ultimately, the clinical interpretation is not always binary, that is, a mutation was detected and therefore is matched to treatment, or a mutation was not detected and therefore no treatment is recommended. To this end, in the following, we take *ESR1* testing as a use case and provide example profiling results and decision‐making thought processes to demonstrate the intricacies of any issues that may arise in the single biomarker indication setting (Fig. 4). Here, we cover six cases of patients with mBC using a liquid biopsy‐based CGP panel (77‐gene panel, assay limit of detection: VAF 0.5%) for the indication of *ESR1* mutation testing for treatment with SERDs.

**Fig. 5:**
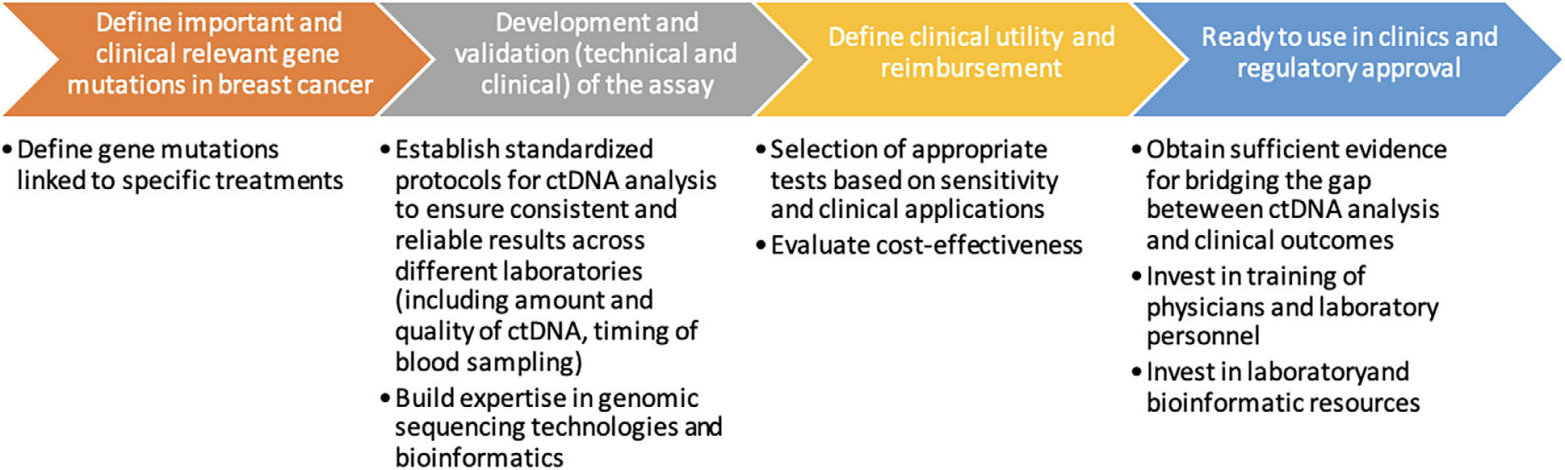
Key steps for integrating liquid biopsy assays into routine clinical workflows (adapted from [50]).

Considering the presumed future increase in molecular alterations, it can be assumed that NGS will reduce the financial burden of testing. Regarding the assay type, targeted sequencing approaches offer the benefit of being more cost‐effective, since only loci of interest or rather clinically relevant hotspot mutations are analyzed, considering that a large part of the genome is still undruggable [138]. Thus, it might be easier to push the reimbursement of liquid biopsies that are directly linked to specific treatments. With an emphasis on the value of molecular characterization for treatment decisions in breast cancer, we finally provide a potential roadmap depicting this integration into clinical practice in Fig. 5.

## 10 Conclusions

Liquid biopsy, particularly the analysis of ctDNA, is under active investigation. This field offers a wide range of applications, from early detection to monitoring of metastatic disease, as well as selection of mutation‐directed therapies [139, 140, 141]. Minimally invasive ctDNA testing, compared to conventional tissue‐based molecular analyses, not only allows for multiple sampling but also enables the visualization of intricate subclonal variations within the spreading tumor. These real‐time insights into tumor dynamics and genetic alterations facilitate personalized therapy selection and enable early and intensified intervention or therapy de‐escalation strategies, with additional psychosocial impact on patient quality of life.

Some of these applications have become routine in clinical practice, such as the analysis of the *PIK3CA* mutational status, which can be performed based on both tissue and plasma samples. In view of this breakthrough and results from ESR1 trials, the 2023 ASCO guideline update strongly recommends *ESR1* ctDNA‐based testing due to its superior sensitivity in the detection of subclonal mutations that have been acquired over time. It also suggests retesting upon disease progression, which demonstrates how the non‐invasive nature of this approach can transform standard clinical practices.

## Conflict of interest

E.V. Klocker received personal fees/travel support from AstraZeneca, DaiichiSankyo, EliLilly, Gilead, Novartis, Roche, and PierreFabre. S. Hasenleithner is a Member of the Advisory Board of CureMatch. R. Bartsch. reports an advisory Role: AstraZeneca, Daiichi, Eisai, Eli‐Lilly, Gilead, Gruenenthal, MSD, Novartis, Pfizer, Pierre‐Fabre, Puma, Roche, Seagen, Stemline, Lecture Honoraria: AstraZeneca, BMS, Daichi, Eisai, Eli‐Lilly, Gilead, Gruenenthal, MSD, Novartis, Pfizer, Pierre‐Fabre, Roche, Seagen, Research Support: Daiichi, MSD, Novartis, Roche. S.P. Gampenrieder reports personal fees/travel support from MSD, Novartis, AstraZeneca, Lilly, Seagen, Daiichi Sankyo, Gilead, Pfizer, Stemline Therapeutics, Janssen, and research support from Roche, Daiichi Sankyo, Novartis, Pfizer, Caris Life Sciences, Lilly, Seagen, Gilead and AstraZeneca. D. Egle reports personal fees/travel support from Amgen, AstraZeneca, Daiichi‐Sankyo, Gilead, Lilly, Mennarini, MSD, Novartis, Pfizer, Roche, Sandoz, Seagen, Sirius Medical. C. F. Singer reports travel, research and unrestricted grants from Novartis, AstraZeneca, Daiichi, Amgen, Roche, Seagen und Gilead Sciences. G. Rinnerthaler reports Honoraria from Amgen, AstraZeneca, Daiichi Sankyo, Eli Lilly, Gilead, MSD, Novartis, Pfizer, Roche, Seagen, Stemline, BMS; Consulting or Advisory Role: Amgen, AstraZeneca, Daiichi Sankyo, Eli Lilly, Gilead, MSD, Novartis, Pfizer, Pierre Fabre, Roche, Stemline; Travel, Accommodations, Expenses: Amgen, Daiichi Sankyo, Eli Lilly, Gilead, Merck, Pfizer, Roche. M. Hubalek reports personal fees/travel support from Amgen, AstraZeneca, DaiichiSankyo, EliLilly, Menarini‐Stemline. K. Schmitz reports personal fees/travel support from Amgen, AstraZeneca, Bayer, Daiichi‐Sankyo, Diaceutics, Discovery Life Sciences, Incyte, Janssen, Merck, MSD, Novartis, Pfizer, PharmaMar, Roche, Sanofi, Servier, Stemline, Takeda and Targos GmbH. Z. Bago‐Horvath reports no conflicts of interest. A. Petzer reports honoraria/advisory role from Novartis, Amgen, Celgene‐BMS, Sandoz, Janssen, AstraZeneca, Abbvie, Takeda, Sanofi, Kite‐Gilead, Roche, Pfizer, Saegen, Daiichi Sankyo and travel support from Roche, Gilead, Daiichi Sankyo, Janssen, Eli Lilly, Pierre Fabre. S. Heibl reports her role in advisory boards and honoraria from Stemline, Daiichi‐Sankyo, Eli Lilly, Novartis, Amgen, Gilead, Roche. E. Heitzer has received unrelated funding from Illumina, Roche, Servier, Freenome, and PreAnalytiX, and received honoraria from Roche and AstraZeneca for advisory boards, not related to our study. M. Gnant reports personal fees/travel support from Amgen, AstraZeneca, DaiichiSankyo, EliLilly, Menarini‐Stemline, MSD, Novartis, PierreFabre, Veracyte; an immediate family member is employed by Sandoz. M. Balic reports personal fees/travel support from Amgen, AstraZeneca, DaiichiSankyo, EliLilly, Gilead, Menarini‐Stemline, MSD, Novartis, PierreFabre, Seagen, and research support from AstraZeneca, Daiichi, Novartis, Pierre Fabre, Pfizer, and Seagen.

## Author contributions

MG, MB, EVK, SH, EH, and AF contributed to the conception and design. The manuscript was written by AF, EVK, and MB. All authors read and approved the final manuscript.
